# Advances in Corneal Diagnostics Using Machine Learning

**DOI:** 10.3390/bioengineering11121198

**Published:** 2024-11-27

**Authors:** Noor T. Al-Sharify, Salman Yussof, Nebras H. Ghaeb, Zainab T. Al-Sharify, Husam Yahya Naser, Sura M. Ahmed, Ong Hang See, Leong Yeng Weng

**Affiliations:** 1 Department of Electrical & Electronic Engineering, College of Engineering, Universiti Tenaga Nasional, Kajang 43000, Malaysia; n2021@esraa.edu.iq (N.T.A.-S.); pe21016@student.edu.my (H.Y.N.); sura_ahmed1991@yahoo.com (S.M.A.); ong@uniten.edu.my (O.H.S.); ywleong@uniten.edu.my (L.Y.W.); 2 Medical Instrumentation Engineering Department, Al-Esraa University College, Baghdad 10069, Iraq; 3 Institute of Informatics and Computing in Energy, Universiti Tenaga Nasional, Kajang 43000, Malaysia; salman@uniten.edu.my; 4 Biomedical Engineering Department, Al Khwarizmi Engineering College, University of Baghdad, Baghdad 10011, Iraq; nebras@kecbu.uobaghdad.edu.iq; 5 Department of Pharmacy, Al Hikma University College, Baghdad 10052, Iraq; 6 School of Chemical Engineering, University of Birmingham, Edgbaston, Birmingham B15 2TT, UK; 7 Environmental Engineering Department, Mustansiriyah University, Baghdad 10052, Iraq

**Keywords:** ophthalmology, corneal topography, diagnostic models, vision health, corneal asphericity, decision tree and nearest neighbor

## Abstract

This paper provides comprehensive insights into the cornea and its diseases, with a particular focus on keratoconus. This paper explores the cornea’s function in maintaining ocular health, detailing its anatomy, pathological conditions, and the latest developments in diagnostic techniques. Keratoconus is discussed extensively, covering its subtypes, etiology, clinical manifestations, and the application of the Q-value for quantification. Several diagnostic techniques, such as corneal topography, are crucial points of discussion. This paper also examines the use of machine learning models, specifically Decision Tree and Nearest Neighbor Analysis, which enhance the accuracy of diagnosing based on topographical corneal parameters from corneal topography. These models provide valuable insights into disease progression and aid in clinical decision making. Integrating these technologies in medical research opens promising avenues for enhanced disease detection. Our findings demonstrate the effectiveness of Decision Tree and Nearest Neighbor Analysis in classifying and predicting conditions based on corneal parameters. The Decision Tree achieved classification accuracy of 62% for training and 65.7% for testing, while Nearest Neighbor Analysis yielded 65.4% for training and 62.6% for holdout samples. These models offer valuable insights into the progression and severity of keratoconus, aiding clinicians in treatment and management decisions.

## 1. Introduction

The cornea is a dome-shaped structure that serves as a pivotal structure in the refractive and visual functions of the eye. It pivots light to focus on the retina so that a crisp image can be produced. The cornea protects the aqueous chamber, iris, and pupil and acts as a windshield to protect the eye from foreign particles. It is devoid of blood vessels but has nerve endings that contribute to the involuntary reflex of closing the eyelid on touch. The aqueous humor and tears provide nutrients to the cornea. A person’s vision relies on corneal health. Some of the cornea’s key characteristics include having refractive power for clear vision, transparency to allow light in, self-repair to maintain integrity, and a barrier function to prevent mechanical injuries.

The cornea is sensitive to injuries and susceptible to various diseases. Therefore, it is crucial to understand its complex anatomy. Physiology sets the framework for studying the pathologies that affect the cornea [1]. Keratoconus, dry eye syndrome, corneal degenerations, pterygium, corneal dystrophies, corneal neovascularization, and corneal ectasia are some of the cornea disorders, with an emphasis on keratoconus. Keratoconus is one of the most common corneal dystrophies and is characterized by the thinning of the cornea and its outward protrusion. Therefore, the q-value (described as the “aspherical degree”) of the cornea tends to be higher in keratoconic eyes as compared to normal corneas. It is a progressive condition which can be treated with contact lenses at first but may require a corneal transplant if it worsens [2].

Over the years, diagnosing keratoconus and other corneal diseases has become more accurate because of advancements in imaging techniques and through cross-sectional visualization of the cornea. These techniques are vital in early diagnosis and prognosis. Some of the medical technologies used to detect such disorders include slit-lamp examination, keratometry, corneal topography, corneal tomography, Pentacam, wavefront analysis, and corneal biomechanical assessment [3].

There are also many computer applications that are integrated with these technologies. Among these applications are the following: Corneal Tomography Software, Wavefront Analysis Software, Machine Learning and Artificial Intelligence (AI) Programs, Optical Coherence Tomography (OCT) Software, Finite Element Analysis (FEA) Software, and Corneal Biomechanical Assessment Software [4].

Medical assessment techniques help manage various and ambiguous data to make decisions in complicated situations. These techniques are useful in disease diagnosis, analyzing treatment methods, and risk assessment. This enables clinical workers to explore keratoconus comprehensively, therefore enabling better identification strategies and treatment plans.

One of this paper’s objectives is to discuss corneal diseases, focusing on keratoconus, along with the current research on them. By studying recent technologies, researchers can understand their transformative effects on ophthalmology. Also, this paper focuses on the methods that can help ophthalmologists to make clinical decisions.

### 1.1. Anatomy and Physiology of the Cornea

The human eye comprises three distinct layers: sclera, choroid, and retina. The sclera is the outermost layer of dense connective tissue. The anterior portion of the sclera is known as the cornea. The cornea is a transparent avascular tissue that acts as a structural barrier and protects the eye against infections [5]. Along with the tear film, it provides a proper anterior refractive surface for the eye. The cornea contributes to two-thirds of the refractive power of the eye [6].

The cornea is horizontally oval, measuring 11–12 mm horizontally and 9–11 mm vertically. Measurements made using the ORBSCAN II system indicate an average corneal diameter of 11.71 ± 0.42 mm, with slight sexual dimorphism noted: 11.77 ± 0.37 in males compared to 11.64 ± 0.47 in females. The corneal diameter ranged from 11.04 to 12.50 in males and from 10.7 to 12.58 in females [7].

The cornea and the lens help focus the light rays onto the back of the eye (retina). The cells in the retina absorb and convert the light to electrochemical impulses, which are transferred along the optic nerve and then to the brain [8].

The cornea’s main characteristics include the anterior portion of the eye, covering the pupil, iris, and aqueous chamber. This is an extension of the sclera, and is composed of proteins and cells and is devoid of blood vessels. Albumin is the most abundant protein present in the cornea. Aqueous humor and tears nourish this layer. The cornea is sensitive to touch, chemicals, heat, and other similar things. It contains unmyelinated nerve endings. These nerve endings are responsible for the involuntary reflex of closing the eyelid on touch and preventing foreign particles from entering and damaging the eye [9].

### 1.2. Cornea Function

The cornea’s primary function is to refract and focus light, accounting for approximately 60 to 75% of the eye’s focusing capacity, while the lens contributes the rest [10]. The lens changes its shape to focus on objects at varying distances, making it responsible for accommodation. A person’s vision is reliant on the cornea’s functionality. Therefore, the cornea’s health is critical in ensuring optimal visual performance. The corneal epithelium keeps the cornea moist by allowing tears to spread and thus provides clear vision [11].

Like the way a camera’s shutter controls light exposure while filming, the iris and pupil operate in a way to manage light intake. In dim lighting, the pupils widen to allow more light into the eye. A camera lens can mechanically adjust and focus on objects at varying distances. Likewise, the eye’s lens performs the same function. However, it may need assistance from glasses, artificial means, or contact lenses for a more precise focus and clearer vision [12].

There are several key characteristics possessed by a cornea for optimal functionality and integrity like refractive power (the cornea’s curvature bends light to form a sharp retinal image, giving it most of the eye’s refractive power), avascularity (the cornea is transparent because it lacks blood vessels, relying on aqueous humor and tears for nutrients and oxygen), transparency (the cornea’s transparency ensures light passes directly to the retina, crucial for clear vision), sensitivity (the cornea’s dense nerve endings make it highly sensitive, triggering protective reflexes like tearing and blinking), self-repair (the cornea can heal and regenerate quickly, with epithelial cells restoring its integrity and function after injury), and barrier function (the cornea shields the eye from debris, pathogens, and injury, with its outer layer defending against mechanical damage and infections). These attributes help protect the cornea in maintaining clear vision and preserving the delicate structures in the eye [13,14,15,16].

### 1.3. The Most Important Health Conditions That Affect the Cornea

The cornea can be susceptible to some of the following diseases and disorders: keratoconus (a progressive condition characterized by distorted and blurred vision as the cornea gets thin and bulges into a cone shape), corneal infections (the cornea can experience vision loss, pain, and inflammation from infections like herpetic or bacterial keratitis), dry eye syndrome (a condition where tears evaporate quickly or lack lubrication, causing discomfort and corneal inflammation), corneal ectasia (a group of conditions causing the cornea to bulge, such as post-LASIK ectasia, where vision distorts due to corneal weakening and protrusion), suspected keratoconus (a preliminary diagnosis indicating that the cornea may be thinning and bulging, but has not yet reached the definitive stage of keratoconus; further tests are typically needed to confirm the condition and assess its severity), forme fruste keratoconus (a mild, early form of keratoconus that does not yet show the full clinical signs of the condition; it is often diagnosed when there are subtle changes in the cornea that suggest keratoconus, but without the pronounced symptoms or visible bulging seen in more advanced cases), and corneal trauma (the cornea, when injured by chemical burns, scratches, or blunt trauma, can suffer from serious damage and complications). These conditions are just a few of the many diseases and complications the cornea may be susceptible to. To maintain optimal vision and eye health, it is important to acquire a diagnosis and receive treatment from an ophthalmologist in a timely manner. This will not be discussed in this research paper because this study has a focus on keratoconus and its types [17,18,19,20,21,22].

## 2. Keratoconus: Diagnosis, Types, and Diagnostic Parameters

### 2.1. The Q-Value

One of the topographic parameters that is extracted from the topography is the aspherical value (Q-value), which has a certain relationship with the shape of the cornea and is important in the diagnosis of keratoconus.

**Figure 1:**
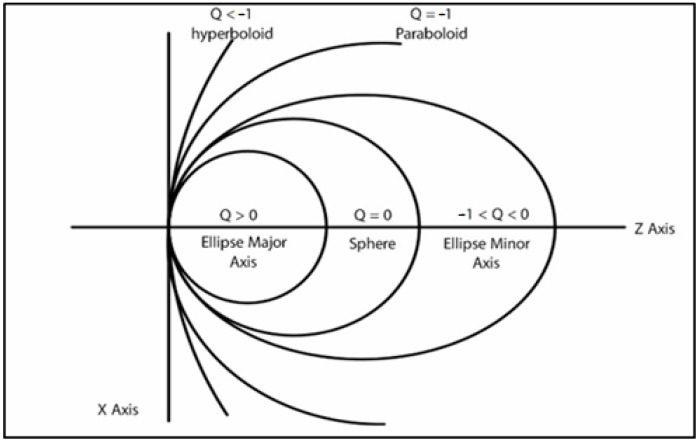
Schematic description of Q-value ranges.

Asphericity (Q-value) describes the rate of radial curvature variation in the corneal quadric surface from its center to the periphery. It is a quantitative topographic indicator of the degree of corneal asphericity, describing the corneal shape and its optical properties [23]. In most cases, corneal asphericity is represented by a prolate ellipse, flattening from the corneal apex to the periphery. A Q value < 0 is considered prolate and a value > 0 is oblate, as shown in Figure 1 [24]. More negative values may suggest keratoconus or hyperopic correction, whereas positive values may suggest myopic correction [25].

The most common diseases that affect the cornea and have a relationship with the q-value are keratoconus, suspected keratoconus, forme fruste keratoconus, and ectasia. Keratoconus is identified by a thin and steep cornea which causes it to bulge. It is a progressive disease characterized by irregular astigmatism. The cornea becomes increasingly asymmetrical, which can be quantified by the q-value [26].

The q-value proves to be high when corneal topography measurements are taken in keratoconus patients. This means there is a greater-than-normal asymmetry in the corneal shape, which causes distorted vision [27].

### 2.2. Keratoconus and Its Types

In the early 19th century, ophthalmologists identified keratoconus as a distinct condition. Mid-20th century advancements in corneal imaging and diagnostics, including detailed corneal mapping in the 1970s, improved understandings of keratoconus. Further developments in imaging techniques, like OCT and Scheimpflug, enhanced diagnostic accuracy through 3D visualization of the cornea.

Keratoconus is a disease that leads to the cornea tending to become thin along with facing a change in its shape. The steepening of the cornea results in it becoming cone-shaped in the temporal-inferior area [28]. Such a problem means that one may start to have blurred vision during their teenage years, and by early adulthood (in some cases, childhood), the problem tends to worsen [29]. A mild–severe distortion may be created due to the change in the curvature of the cornea. These problems are commonly known as near-sightedness and astigmatism. While soft contacts or glasses improve vision but do not solve the problem for most people, some other routes may be needed for other people. A cornea transplant is performed for some people with keratoconus [30].

In terms of the visible differences between a healthy cornea and a keratoconus one, it is understood that an eye with keratoconus has a cornea that thins and slowly bulges towards the outer side and develops into a cone shape. Due to these changes, significant distorted vision, as well as blurriness, may occur in bilateral diseases [31,32].

Keratoconus diagnosis starts with detailed clinical history and careful examination with the aid of a slit lamp. However, using associated data from new technologies to increase diagnostic accuracy is still challenging. Corneal topography with all types of Placido rings, Scheimpflug, 3D corneal tomography, segmental tomography, wavefront analysis, and corneal biomechanical assessments and tests are the most relevant diagnosis methods [33].

The detection of keratoconus is a major concern in the screening of refractive surgical patients since it is known that its presence weakens the corneal stroma and can lead to iatrogenic ectasia. Clinical keratoconus can be reliably detected using corneal topography and Scheimpflug imaging, providing a means to identify keratoconus in its earliest stages. This approach has been extensively studied. Several terms have been put forward to describe this condition, including keratoconus, suspected keratoconus, ectasia, and forme fruste keratoconus. While these designations have been used interchangeably, doing so has led to problems in understanding the natural history of keratoconus [34].

Forme fruste keratoconus is a subclinical disease and is not a variant of keratoconus. Although clinicians use many other terms such as mild keratoconus, early keratoconus, and subclinical keratoconus, their exact meanings and applications are less certain. Forme fruste keratoconus (FFKC), recognized as the earliest stage of keratoconus, is frequently cited in the literature under various terminologies, including forme fruste keratoconus (FFKC) [35]. It is characterized by the absence of abnormal findings in both slit-lamp examinations and Scheimpflug-based corneal topography. In cases of clinical keratoconus, there is a consensus that FFKC represents the healthier eye of the patient, devoid of the clinical signs associated with manifest keratoconus [36,37].

Corneal ectasia refers to a group of non-inflammatory disorders of the eye that involve the bilateral thinning of the cornea. Keratoconus is a specific type of corneal ectasia in which the cornea thins and weakens, leading to bulging and distortion. Corneal ectasia is a rare but serious complication resulting from vision correction procedures such as laser-assisted in situ keratomileusis (LASIK) and photorefractive keratectomy (PRK). It is associated with worsening vision and is marked by progressive corneal bulging and thinning [38].

The term “keratoconus suspect” should be specifically applied to corneas exhibiting distinct topographic changes, particularly those lacking evidence of keratoconus in the fellow eye. It typically displays localized abnormal steepening, often in the inferior region but possibly also in the central or, rarely, superior regions. This may manifest as an asymmetrical, truncated, or skewed-axis bowtie pattern [34].

Keratoconus can affect the q-value of the cornea. In keratoconus, the cornea gradually steepens, resulting in a cone-shaped bulge which can affect the asphericity. As a result, the Q-value increases in keratoconic eyes, becoming more positive compared to the typically negative values in normal corneas.

Using the q-value can aid in determining whether any region of the cornea has steeped or is asymmetrical. Higher asymmetry is indicated by a higher q-value which is also characterized by irregular astigmatism in the cornea.

### 2.3. Keratoconus Diagnostic Methods and Technologies

The methods of diagnosis and examination of the cornea are diverse and numerous. Table 1 presents information on some medical and engineering methods for the identification of keratoconus. 

If these diagnostic techniques are used together, they help detect keratoconus in its early stages and allow for timely treatment and the prevention of vision disabilities [27].

## 3. Methods

Keratoconus is characterized by the thinning of the cornea and irregularities of the cornea’s surface. There are many methods used for the purpose of obtaining diagnoses of the keratoconus and its degree and type. Some of the most common ways, in which strong associations that are useful for diagnoses have been obtained, are summarized in the following subsections.

### 3.1. Decision Tree

A Decision Tree is a supervised learning algorithm utilized for both classification and regression tasks. This method constructs a tree-based model to classify cases into groups or predict values of a dependent (target) variable based on independent (predictor) variables. It includes tools for validation in both exploratory and confirmatory classification analysis. The Decision Tree method was selected due to its foundation in classification principles, making it a key algorithm in machine learning for predicting target variable values from several input features [46]. This approach is particularly relevant for this work, as it has proven to be effective in yielding accurate predictions, unlike other classification methods that did not perform as well.

Table 2 below shows the input parameters used by the Decision Tree classification algorithm.

### 3.2. Nearest Neighbor Analysis

Nearest Neighbor Analysis is a method for classifying cases based on their similarity to other cases. In machine learning, it is used to recognize patterns of data without requiring an exact match to stored patterns or cases. Similar cases are close to each other, while dissimilar cases are distant. The distance between cases measures their dissimilarity, and the classifications of the most similar cases—the nearest neighbors—are tallied. The new case is placed into the category that contains the greatest number of nearest neighbors [48]. The number of nearest neighbors to examine is specified by the value k which is the number of input data.

This theory utilizes a forecasting approach that relies on analyzing the nearest data points to make accurate decisions. It also incorporates elements of classification and prediction.

## 4. Results and Discussion

### 4.1. Quantitative Assessment of Corneal Disorders

In this research paper, 491 patients were used to carry out a research study at the Al-Amal Center in Baghdad to investigate the impact of keratoconus.

- Of the patients, 226 showed normal ocular conditions (N).
- Of the patients, 64 presented keratoconus (K). The diagram below depicts the numerical relationship between the q-value and patients with keratoconus.
- Of the patients, 70 were diagnosed with forme fruste keratoconus (FFKC), cited in this research paper as (F), showing a correlation between the q-value and their condition.
- Further research in this work found 22 patients with ectasia, cited in this research paper as (A), and 109 were found to have subclinical or suspected keratoconus (SSKC), cited in this research paper as (S).

### 4.2. Decision Tree 

Using SPSS V.23 software, the Decision Tree classification was calculated based on input parameters that strongly influence the model (Astig, Patchy apex, K-Max, Bowties shape, k1, k2, km, pupil center) to diagnose pathological conditions (N, S, F, K, and A). The optimal data division was found to be 86% for the training sample and 14% for the testing sample.

**Figure 2:**
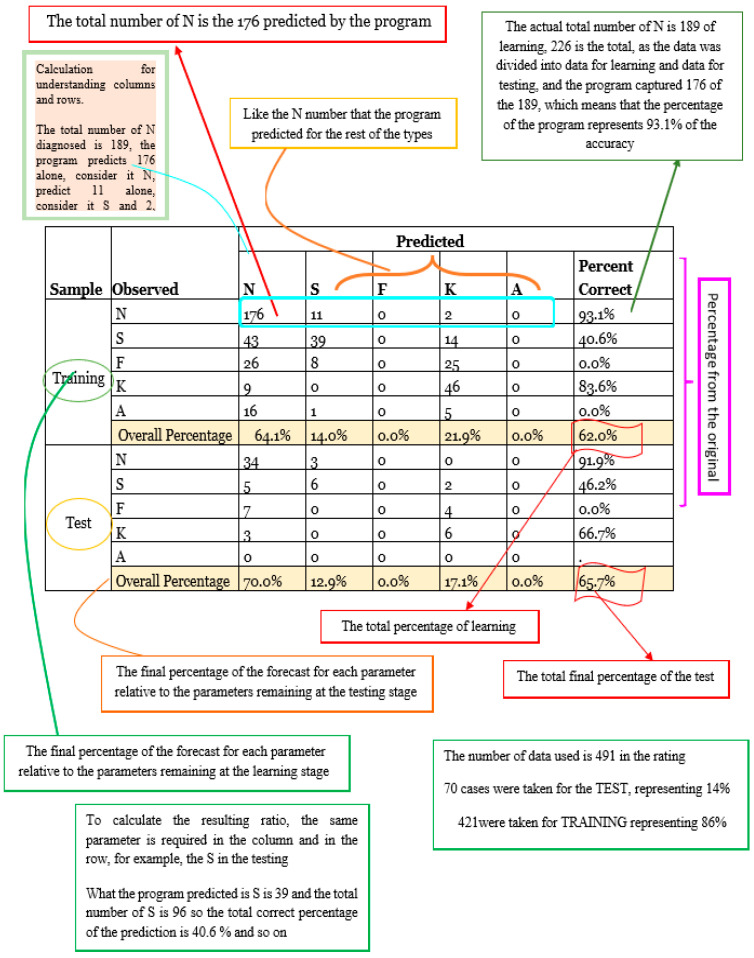
Summary of classification for DT.

**Figure 3:**
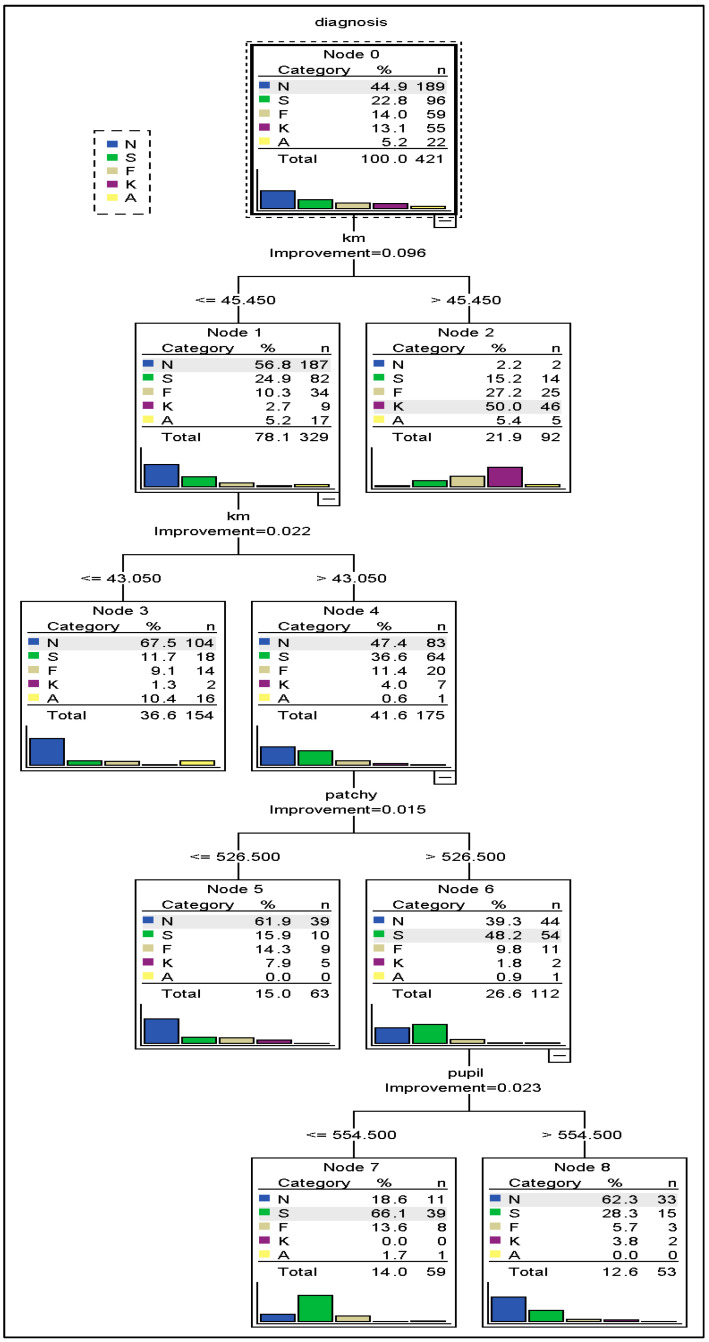
Tree diagram for training sample.

**Figure 4:**
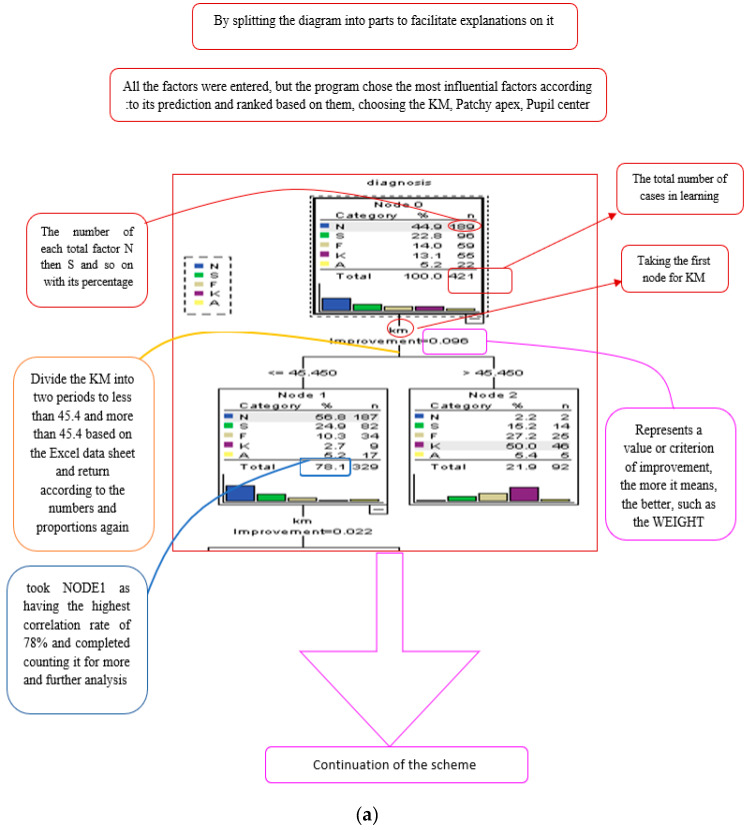

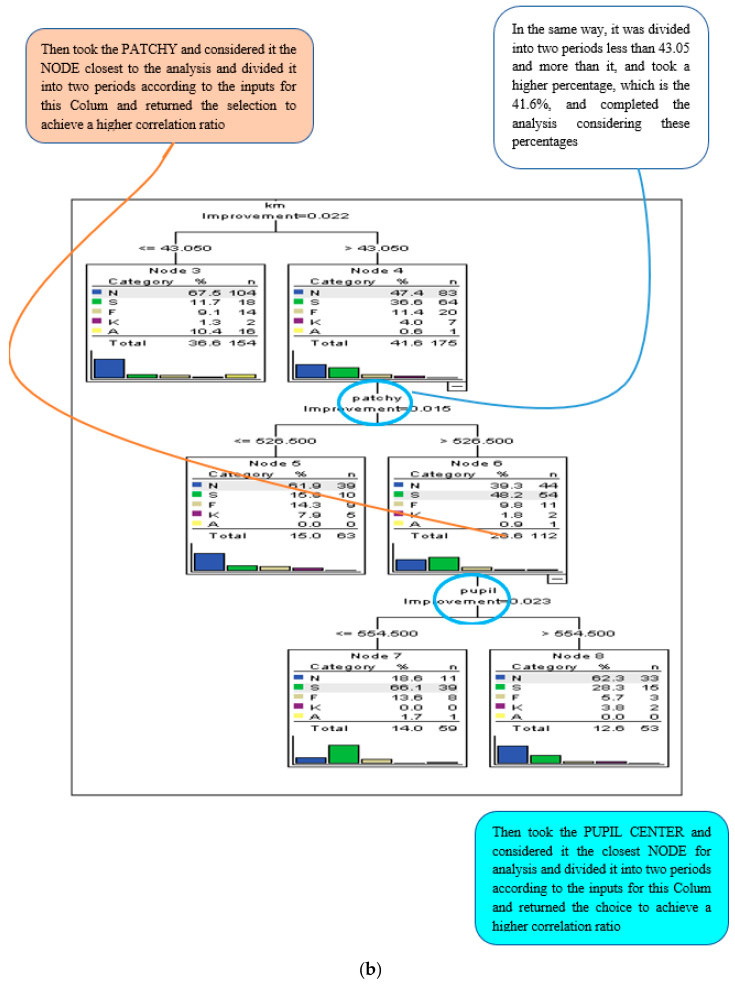

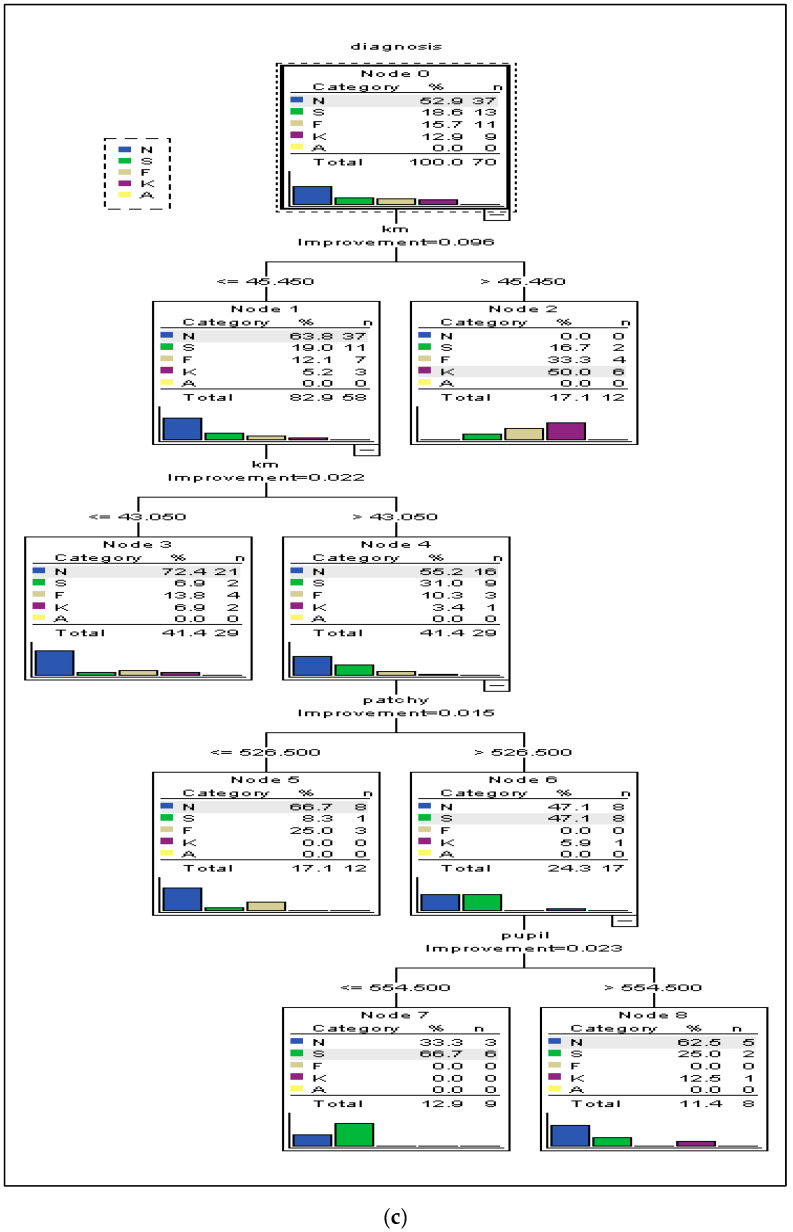
(**a**–**c**): Tree diagram for testing sample.

Figure 2 displays the percent correctness of the classification diagnosis for training and testing samples, which are 62% and 65.7%, respectively. Figure 3 and Figure 4 illustrate the classification process using a tree diagram for both the training and testing samples, respectively.

### 4.3. Nearest Neighbor Analysis

Although the previous theory showed a stronger correlation with a 65% success rate, the current theory achieved a slightly lower correlation of 62%.

**Figure 5:**
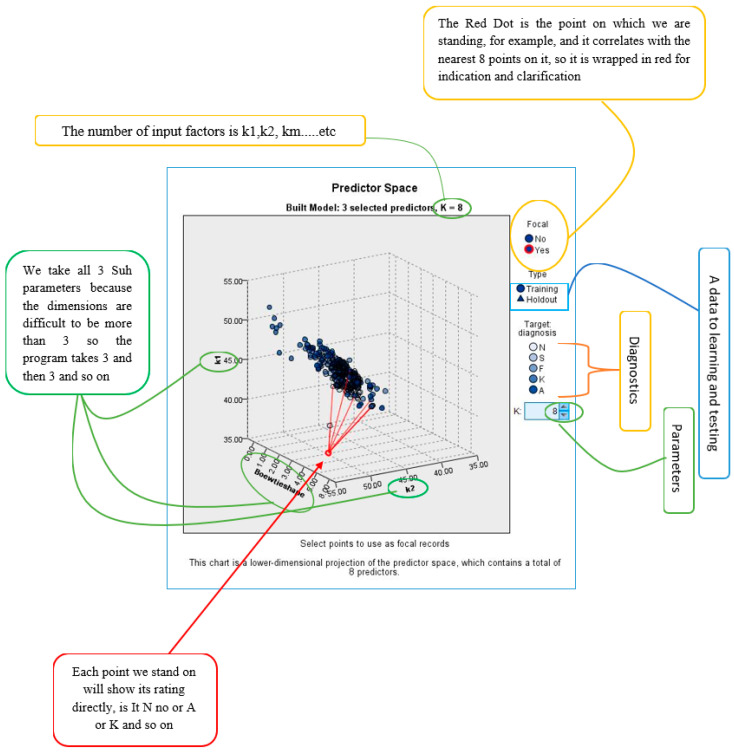
Model viewer for training and holdout samples.

Using SPSS V.23 software, the classification was calculated based on input parameters (astig, patchy apex, K-Max, Boewtie shape, k1, k2, km, pupil center) to diagnose pathological conditions (N, S, F, K, and A). The optimal data division was found to be 68.4% for the training sample and 31.6% for the holdout sample. Figure 2 displays the percent correctness of the classification diagnosis for training and holdout samples, which are 65.4% and 62.6%, respectively. Figure 5 shows the model viewer diagram for training and holdout samples, indicating the closest eight points and distance from the predictor point. For example, Table 3 shows the nearest neighbor and distances for focal point 180, through which the prediction is calculated for diagnosis.

The structure and presentation of Table 3 align closely with those of Figure 2. Both tables employ a similar analytical framework to ensure consistency in the comparison of results. However, the primary distinction lies in the theoretical basis underlying each table. Table 3 is grounded in a different theoretical model, which is essential for showcasing the variation in outcomes when applying alternative methodologies. This parallel approach enables a clearer understanding of how different theories influence the diagnostic performance metrics, reinforcing the validity and robustness of the findings of this paper.

Table 3 presents the results of the Nearest Neighbor Analysis (NNA) model applied to the dataset for corneal diagnostics. Unlike Figure 2, which utilized the Decision Tree algorithm, Table 3 is based on a different theoretical approach—Nearest Neighbor Analysis classification. This method classifies cases based on the proximity of input variables to previously classified cases.

In analyzing the data, we calculated the nearest neighbors for each focal point, using a set of key parameters such as corneal curvature (K1, K2), astigmatism, and corneal apex measurements. These parameters were inputted into the NNA model to identify patterns and similarities between the focal points and neighboring data points.

**Figure 6:**
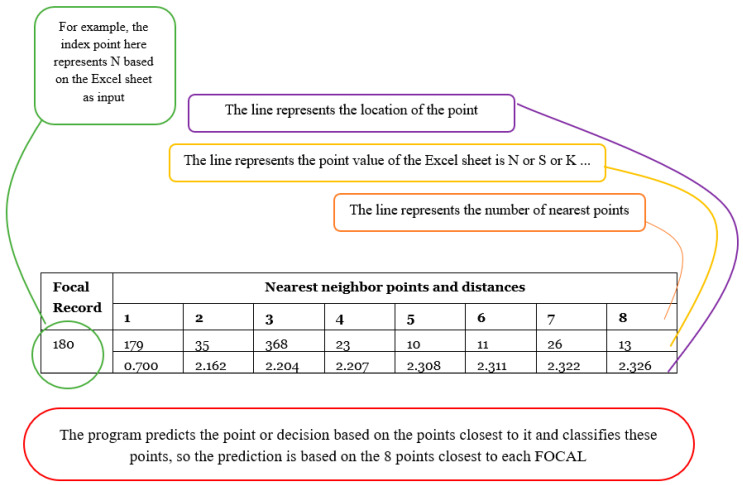
k Nearest neighbor and distances.

The performance of the NNA model is quantitatively assessed by its classification accuracy for the training and holdout samples, with results presented in Table 3. The model achieved 65.4% accuracy for the training set and 62.6% for the holdout sample. Figure 6 presents the k Nearest neighbor and distances. These findings demonstrate the model’s robustness in diagnosing pathological conditions based on corneal parameters, particularly when compared to traditional methods. 

### 4.4. Comparison of Machine Learning Models and Traditional Diagnostic Methods for Keratoconus

To evaluate the performance of machine learning models, such as the Decision Tree and Nearest Neighbor Analysis (NNA), in diagnosing keratoconus, we compared their specificity, sensitivity, and overall accuracy with those of traditional diagnostic methods like keratometry and corneal topography.

Traditional methods like keratometry and corneal topography are widely used for detecting keratoconus by measuring corneal curvature and surface irregularities. These methods typically yield high specificity (up to 90%) in advanced cases but can be less sensitive in detecting early or subclinical keratoconus. In contrast, machine learning models demonstrated improved sensitivity in identifying early stages of keratoconus by analyzing complex patterns in the data. For example, the Decision Tree model achieved a sensitivity of 83.6% for keratoconus classification, outperforming traditional methods, which can sometimes fail to detect subtle corneal abnormalities in the early stages of the disease.

In terms of overall accuracy, the machine learning models provided comparable or better results than traditional diagnostic tools. The Decision Tree achieved an overall accuracy of 62% for training samples and 65.7% for testing samples, while Nearest Neighbor Analysis showed 65.4% accuracy for training and 62.6% for holdout samples. Traditional methods like corneal topography and keratometry, while effective in later stages of keratoconus, typically show lower accuracy when it comes to predicting disease progression or diagnosing ambiguous cases.

Machine learning models offer the ability to process and analyze a wide range of parameters simultaneously (e.g., K1, K2, astigmatism, corneal asphericity). This allows them to detect subtle patterns and irregularities that traditional methods may overlook, particularly in early or subclinical keratoconus. Moreover, machine learning algorithms like Decision Trees and NNA adapt to new data, improving their accuracy over time, whereas traditional diagnostic methods rely on predefined clinical thresholds and may require manual interpretation, which introduces variability.

Several studies have indicated the advantages of machine learning models in ophthalmology. For example, a study by Romero-Jiménez et al. [28] found that machine-learning-based corneal topography models demonstrated a higher sensitivity for detecting early keratoconus compared to conventional diagnostic tools. Similarly, Alió et al. [3] reported that incorporating machine learning algorithms in corneal diagnostics significantly enhanced the accuracy of disease classification, particularly in complex cases.

While machine learning models have demonstrated promising results, it is important to note that they may be limited by the quality and size of the datasets they are trained on. Traditional methods, on the other hand, are well established and trusted by clinicians due to their decades-long use in practice. Further validation and larger datasets are required to fully establish the superiority of machine learning models across all stages of keratoconus diagnosis.

## 5. Challenges and Limitations in Clinical Application of Machine Learning Models

### 5.1. Data Availability and Quality

Machine learning models rely heavily on the quality and quantity of data they are trained on. In clinical settings, the availability of large, well-labeled datasets is often a significant limitation. Medical datasets are typically constrained by privacy concerns, variability in recording practices across institutions, and inconsistencies in the types of diagnostic measurements captured. In the context of corneal diagnostics, limited access to comprehensive corneal topography data can hinder the performance of models, particularly when detecting less common conditions such as subclinical or early-stage keratoconus. To address this, robust anonymization protocols and collaborative data-sharing frameworks need to be developed, facilitating access to high-quality datasets while maintaining patient confidentiality.

### 5.2. Need for Extensive Training Datasets

For machine learning models to generalize well across different patient populations, they require large and diverse datasets. This is particularly critical in ophthalmology, where the variations in corneal shape and disease progression can be subtle and patient-specific. If training datasets are not sufficiently representative of different demographics—such as age, ethnicity, or severity of keratoconus—there is a risk that the models will overfit to specific cases and fail in broader clinical applications. To mitigate this, the development of large-scale, multi-center datasets is essential, ensuring that models can accurately classify and predict conditions across diverse populations.

### 5.3. Integration into Clinical Workflows

One of the most significant challenges in adopting machine learning models in clinical settings is the complexity of integrating them into existing diagnostic workflows. Many clinical practices rely on traditional tools and techniques, such as corneal topography and keratometry, which are widely trusted by ophthalmologists. Introducing machine learning algorithms, such as Decision Trees or Nearest Neighbor Analysis, into these workflows may face resistance due to concerns about interpretability and trustworthiness. Clinicians require tools that not only provide accurate predictions but also explain their decision-making processes in a transparent and clinically relevant manner.

To ensure widespread adoption and reliability in diagnosing corneal diseases, the following strategies can be implemented:

1. Model transparency: Machine learning models must offer clear explanations for their predictions, ensuring that clinicians can understand the rationale behind diagnostic recommendations. Developing models with explainable AI (XAI) features can help build trust among healthcare professionals.
2. User-friendly interfaces: The success of machine learning integration depends heavily on creating interfaces that are easy to use and integrate seamlessly into existing clinical software. Simplified workflows and intuitive outputs are critical for ensuring adoption.
3. Rigorous validation and testing: Before being widely implemented, these models must undergo extensive validation using diverse datasets to ensure reliability across different patient populations. Continuous evaluation and updates based on new data will be essential to maintain model accuracy.
4. Training and education: Clinicians need to be trained on how to effectively use machine learning tools. Educational initiatives should focus on how these models enhance decision making rather than replacing the clinician’s expertise.
5. Clinical trials and pilot programs: Initial pilot programs and clinical trials can demonstrate the efficacy and safety of these models in practice. Successful early implementations will encourage broader adoption within the clinical community.
6. Collaborative approach: A collaborative approach between data scientists, clinicians, and regulatory bodies is crucial. This ensures that machine learning models meet both clinical and ethical standards, enhancing their acceptance in clinical settings.

These strategies aim to address the challenges that arise during the integration of machine learning models into routine clinical practice, ensuring their effective use in diagnosing corneal diseases such as keratoconus.

### 5.4. Bias and Ethical Considerations in Machine Learning Predictions

Machine learning models are susceptible to biases that can arise from imbalanced datasets, which may overrepresent certain conditions or demographic groups. In the case of corneal disease diagnostics, if a dataset is skewed toward more severe cases of keratoconus or contains fewer examples of early-stage disease, the model may underperform in detecting the condition at its onset. Additionally, if the training data do not include diverse patient populations, there is a risk of misdiagnosis or less accurate predictions for underrepresented groups. These biases raise significant ethical concerns, particularly in medical settings where diagnostic accuracy directly impacts patient outcomes. To address this, it is crucial to implement rigorous validation protocols, including cross-validation across diverse datasets, and continually update models as new data become available. Moreover, regulatory frameworks should be established to ensure that machine learning models in healthcare meet ethical standards, including the protection of patient privacy, fairness in diagnosis, and accountability for model decisions.

### 5.5. Ethical Considerations and Patient Data Privacy in Machine Learning for Medical Diagnostics

The integration of machine learning in medical diagnostics presents significant ethical challenges, particularly around the use of patient data. Machine learning models are data-driven, requiring vast amounts of patient information to train and improve accuracy. It is essential to ensure that patient data are used responsibly, with strict adherence to ethical guidelines that prioritize patient consent and transparency. Patients must be informed about how their data will be used, and they should have the ability to opt in or out of data-sharing initiatives without compromising their access to care. Failure to uphold these ethical standards could lead to breaches of trust between healthcare providers and patients, undermining the potential benefits of machine learning in diagnostics.

To protect patient privacy, anonymization of data is critical when using machine learning models in clinical settings. Anonymization involves removing or obfuscating personally identifiable information (PII), such as names, addresses, and medical record numbers, ensuring that data cannot be traced back to individual patients. This process helps mitigate the risk of data misuse or breaches, while allowing for the continued development of machine learning algorithms. However, achieving effective anonymization is complex and must balance the need for data integrity with privacy protection. Advanced techniques, such as differential privacy, can be employed to enhance the security of patient data while still allowing machine learning models to learn from them.

Machine learning models can introduce or exacerbate biases if the training data are not representative of the population being diagnosed. In medical diagnostics, such biases may lead to unequal treatment or misdiagnosis for certain demographic groups, particularly those who are underrepresented in the training data. For example, a model trained primarily on data from younger patients may underperform when diagnosing conditions in older populations. Addressing these biases requires the careful curation of diverse and representative datasets, as well as ongoing monitoring of model performance across different population groups. Failure to mitigate bias could have serious ethical implications, potentially leading to disparities in healthcare delivery.

To ensure patient privacy and the ethical use of machine learning in healthcare, adherence to regulatory frameworks such as the General Data Protection Regulation (GDPR) in Europe and the Health Insurance Portability and Accountability Act (HIPAA) in the United States is crucial. These regulations provide guidelines on how patient data should be collected, processed, and stored, with a particular focus on protecting sensitive health information. Compliance with these frameworks helps safeguard patient privacy while enabling the use of machine learning in medical diagnostics. Additionally, regulatory oversight ensures that data security measures, such as encryption and access controls, are in place to prevent unauthorized access or data breaches.

## 6. Conclusions

This research paper seeks to cover the relationships between various parameters affecting medical decision making for corneal diseases, with a particular focus on the most critical types of keratoconus. This represents a significant advancement in diagnostic technologies that have transformed ophthalmic practices. By thoroughly examining keratoconus and its subtypes, the paper underscores the crucial role of early diagnosis and effective management in preserving optimal visual health.

A comprehensive analysis of the eight parameters included in the analysis and their relationship with corneal diseases, in particular keratoconus, emphasizes the decisive role of accurate diagnostic criteria in assessing the development of the disease. The integration of advanced diagnostic techniques, such as corneal topography, wavefront analysis, and corneal tomography, coupled with the use of sophisticated software and machine learning algorithms, has significantly enhanced the accuracy of detection and management of corneal disorders.

The Decision Tree and Nearest Neighbor Analysis methods, as applied in this study, demonstrate the effectiveness of machine learning models in classifying and predicting conditions based on key corneal parameters. In Decision Tree, the percent correctness of the classification diagnosis for training and testing samples are 62% and 65.7%, respectively; meanwhile, in Nearest Neighbor Analysis methods, the percentages of correctness of the classification diagnosis for training and holdout samples are 65.4% and 62.6%, respectively. These models provide valuable insights into the progression and severity of diseases like keratoconus, aiding clinicians in making informed decisions regarding treatment and management.

The advancement of diagnostic and analytical techniques promises significant progress in ophthalmology. Leveraging these innovations can support healthcare professionals with decision making in diagnostic predictions, improving early detection, personalizing treatment plans, and ultimately enhancing patient outcomes. Continuous scientific research and the development of corneal health diagnostic tools paves the way for a future where precision medicine markedly enhances quality of life for individuals with corneal diseases.

## Figures and Tables

**Table 1:** Some corneal diagnostic techniques.

The Technique	Definition
Keratometry	The measurement of the cornea’s curvature, particularly the central corneal curvature. One of the key findings of keratoconus is a steepened corneal curvature, especially in the inferior region [39,40].
Corneal topography	A non-invasive imaging technique that charts the corneal surface curvature. The test is effective in diagnosing keratoconus as it provides comprehensive data on the elevation, shape, and curvature of the cornea [27,41].
Optical coherence tomography (OCT)	A non-invasive imaging technique that uses light waves to capture high-resolution cross-sectional images of biological tissues [42].
Corneal tomography	Optical coherence tomography (OCT) and Scheimpflug imaging help produce corneal tomography images. Corneal tomography is another three-dimensional imaging technique that evaluates the cornea’s morphological features, such as its thickness and elevation, to help detect keratoconus [27,43].
Pentacam	Pentacam is an analytical device that uses a type of corneal tomography system called Scheimpflug imaging to identify any alterations in corneal thickness, curvature, and elevation to help classify keratoconus [27,44].
Corneal pachymetry	Measures corneal thickness, which is critical in diagnosing glaucoma and evaluating corneal health [45].

**Table 2:** The input parameters [27,47].

Symbol	Definition
K_1_	The cornea’s horizontal curvature power in the middle 3 mm circle, represented in diopters.
K_2_	Vertical curvature power of the cornea in the central 3 mm, expressed in diopters.
Kmax	The cornea’s max curvature power in the center 3 mm, represented in diopters.
Bowtie shape	Topographic shape patterns.
A_STIG_	The degree of divergence between the two curvature radii of the middle 3 mm on the front cornea surface, i.e., the amount of corneal astigmatism.
Patchy apex	The apical thickness of the cornea. The apex is assumed to be the origin of the coordinates by the computer, with “X” representing the horizontal and “Y” representing the vertical. As a result, the value zero is displayed in both squares of patchy apex coordinates.
Pupil center	Corneal thickness at the center of the pupil. The “X” and “Y” coordinates indicate the distance between the pupil center and the apex. Because the pupil center is often displaced super temporally when dilated, the two coordinates alter depending on pupil medriasis or miosis. This is critical in decent rationing, also known as offset pupil.

**Table 3:** Summary of classification for NNA.

Sample	Observed	Predicted					
		N	S	F	K	A	Percent Correct
Training	N	145	14	1	0	1	90.0%
	S	40	32	1	0	0	43.8%
	F	19	10	14	1	0	31.8%
	K	3	5	8	26	0	61.9%
	A	8	1	4	0	1	7.1%
	Overall Percentage	63.9%	18.4%	8.3%	8.0%	0.5%	65.4%
Holdout	N	59	5	1	0	0	58.3%
	S	12	7	5	0	0	20.8%
	F	12	7	5	0	0	20.8%
	K	4	2	4	12	0	54.5%
	A	6	0	2	0	0	0.0%
	Overall Percentage	58.7%	22.5%	10.9%	7.7%	0.0%	62.6%

## Data Availability

The data supporting the findings of this study are not publicly available due to restrictions related to ongoing analyses and further research development. For any inquiries, please contact the corresponding author.
